# Mn^2+^ Ion‐Mediated Zn Dendrite Suppression and Reversible Stripping in Aqueous Zn‐Ion Batteries as Revealed by In Situ Liquid‐Cell TEM

**DOI:** 10.1002/advs.76489

**Published:** 2026-07-16

**Authors:** Zhenhuan Chen, Dasun P. W. Guruge, Kudachchige Asanga G. de de Alwis, Kaveendra V. Maduwantha, Konstantin L. Firestein, Joseph F.S. Fernando, Chao Zhang, Dmitri V. Golberg

**Affiliations:** ^1^ School of Chemistry and Physics and Centre for Materials Science Queensland University of Technology (QUT) Brisbane Queensland Australia; ^2^ Central Analytical Research Facility and Centre for Materials Science Queensland University of Technology (QUT) Brisbane Queensland Australia; ^3^ Centre for Microscopy and Microanalysis The University of Queensland St Lucia Queensland Australia

**Keywords:** aqueous zinc ion battery, dendrite suppression, electrolyte engineering, In situ liquid‐phase TEM, interfacial deposition

## Abstract

Achieving uniform Zn deposition and its reversible dissolution at the anode remains a major challenge in aqueous zinc‐ion batteries (AZIBs), where dendrite growth and interfacial instability limit the cycling life. Herein, we employ in situ electrochemical liquid‐phase transmission electron microscopy (EC‐LPTEM) to mimic a battery operation and directly visualize Zn electrodeposition in the presence of MnSO_4_ in an aqueous ZnSO_4_ electrolyte. Real‐time observations reveal that Mn^2+^ ions regulate interfacial nucleation, suppress dendritic elongation, and promote uniform plating, while facilitating nearly complete Zn dissolution during stripping. Post‐deposition analyses using transmission electron microscopy (TEM), selected area electron diffraction (SAED), and scanning transmission electron microscopy–energy dispersive X‐ray spectroscopy (STEM‐EDS) show that Mn^2+^ ions are incorporated into chemically labile Zn–Mn–O–S interfacial deposits. Ex situ electrochemical measurements confirm that Mn^2+^ ions improve Zn anode stability and extend cycling performance. These findings provide direct nanoscale insights into additive‐regulated Zn deposition and highlight Mn^2+^ ion‐mediated interfacial modulation as an effective strategy for controlling Zn deposit morphology and enhancing AZIB durability.

## Graphical abstract

In situ EC‐LPTEM directly visualizes how Mn^2+^ ions regulate Zn electrodeposition and dissolution in aqueous electrolytes. Mn^2+^ suppresses heterogeneous growth and dendritic elongation while promoting more reversible stripping. These findings reveal additive‐regulated interfacial processes during Zn deposition and dissolution and provide guidance for the design of durable aqueous zinc‐ion batteries.

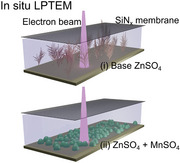

## 1 Introduction

Aqueous zinc‐ion batteries (AZIBs) have attracted extensive attention for large‐scale energy storage owing to their intrinsic safety, low cost, and environmental compatibility [1, 2]. However, their practical deployment remains hindered by low Coulombic efficiency, limited cycling stability, and insufficient energy density [1, 3, 4]. These challenges mainly originate from the inhomogeneous deposition of Zn on the anode, which promotes parasitic reactions such as corrosion and hydrogen evolution, induces internal short circuits through uncontrolled dendritic Zn growth, and generates electrochemically inactive “dead Zn”, ultimately resulting in irreversible capacity loss [5, 6, 7, 8, 9]. Consequently, controlling Zn nucleation and growth at the electrode–electrolyte interface has become a central challenge for improving the electrochemical performance and long‐term durability of AZIBs [10].

In conventional AZIB systems, aqueous ZnSO_4_ electrolytes are widely used, where Zn^2+^ ions typically coordinate with six H_2_O molecules to form the classical solvation structure [Zn(H_2_O)_6_]^2+^ [11, 12, 13]. The strong hydration of Zn^2+^ ions leads to sluggish desolvation kinetics during Zn deposition, often resulting in uneven plating and dendritic growth, which further aggravates parasitic reactions and interfacial passivation [14, 15, 16]. Consequently, extensive electrolyte engineering efforts have been devoted to modulating Zn deposition behavior.

Among various approaches, metal salt additives have attracted increasing interest owing to their wide availability, thermal stability, and capability to modulate the electrolyte environment [17]. Two primary mechanisms have been proposed to regulate Zn deposition. First, foreign metal cations can compete with Zn^2+^ ions for coordination with H_2_O molecules, thereby modifying the Zn^2+^ solvation structure. Second, additional cations can redistribute the interfacial electric field near the Zn surface, promoting more uniform Zn deposition [18, 19, 20].

Representative examples include Al^3+^ ions from Al_2_(SO_4_)_3_, which alter the Zn^2+^ solvation structure and screens the interfacial electric field to suppress dendrite formation [21, 22, 23]. Similarly, Ce^3+^ ions from CeCl_3_ can form a dynamic electrostatic shielding layer near Zn protuberances, promoting uniform Zn deposition [24]. Other cations, including Gd^3+^, K^+^, Rb^3+^, and Na^+^, have also been reported to mitigate dendritic growth and side reactions through electrostatic shielding effects [25, 26, 27, 28, 29]. In addition, certain additives, such as LiCl, Pb^2+^, Ni^2+^, Sn^2+^, and In^3+^, can regulate Zn dissolution and deposition processes, thereby enhancing anode stability [30, 31, 32, 33]. Even when direct dendrite suppression is not clearly demonstrated, additives such as Co^2+^ and Mg^2+^ ions have been shown to improve cycling stability and overall electrochemical performance [34, 35].

Among these metal salt additives, MnSO_4_ has received particular attention in ZnSO_4_‐based AZIBs, especially in systems employing MnO_2_ cathodes. Mn^2+^ ions can regulate the Mn dissolution equilibrium from MnO_2_ and facilitate reversible MnO_x_ deposition/dissolution, thereby improving cathode stability [36, 37, 38, 39, 40]. Beyond its cathode‐related effects, Mn^2+^ may also influence Zn deposition at the anode by interacting with interfacial byproducts formed during electrodeposition [41, 42]. Owing to its lower reduction potential than Zn^2+^, Mn^2+^ can mitigate dendrite formation during charging via an electrostatic shielding mechanism [10, 18, 43].

Despite these advances, most investigations rely on post‐plating characterization, such as scanning electron microscopy (SEM), which cannot capture dynamic deposition processes. Although in situ optical microscopy and synchrotron X‐ray imaging have been used to probe Zn deposition behavior in the presence of certain additives [30, 31], these approaches lack the spatial resolution required to resolve early‐stage nucleation and nanoscale growth processes. As a result, direct visualization of how dissolved metal salt additives influence Zn nucleation, interfacial growth, and morphological evolution at the sub‐micron or nanoscale remains scarce. However, such insight is essential for elucidating the underlying mechanisms and guiding rational electrolyte design.

In this context, in situ electrochemical liquid‐phase transmission electron microscopy (EC‐LPTEM) provides a powerful platform for probing Zn electrodeposition with high spatial resolution. EC‐LPTEM enables direct observation of early‐stage Zn nucleation and growth under electrochemical conditions, offering unique insights into nanoscale deposition dynamics in the presence of electrolyte additives [44, 45]. Previous EC‐LPTEM studies have investigated Zn plating/stripping behavior in ZnSO_4_‐based electrolytes and revealed that LiCl, Mn^2+^, and CF_3_SO_3_^−^ species can help suppress dendritic Zn growth [46, 47]. Beyond capturing dynamic deposition, EC‐LPTEM is also valuable for characterizing products formed adjacent to the electrode surface, providing insights relevant to the electrode–electrolyte interfacial region. For example, high‐quality 4D scanning transmission electron microscopy (4D‐STEM) has been applied to resolve crystallographic details during Zn deposition [48], while in situ electrochemical liquid‐cell STEM studies have revealed multistep Zn dendrite growth pathways involving nucleation, hexagonal nanosheet formation, nanosheet stacking, and dendrite evolution [49].

Thus, herein, in situ EC‐LPTEM was employed to visualize Zn electrodeposition in a 0.1 M ZnSO_4_ electrolyte with and without a 20 mM MnSO_4_ additive. Real‐time observations show that Mn^2+^ ions regulate interfacial Zn deposition, leading to more uniform Zn plating and enhanced reversibility during dissolution. Post‐deposition transmission electron microscopy (TEM), selected‐area electron diffraction (SAED), and scanning transmission electron microscopy energy dispersive X‐ray spectroscopy (STEM‐EDS) analyses further reveal that deposits formed near the electrode in the presence of Mn^2+^ ions consist of mixed Zn–Mn–O–S species, which are more chemically labile than those in the additive‐free electrolyte. Consistently, ex situ electrochemical measurements of both symmetric and full cells with MnSO_4_‐supplemented electrolytes exhibit more stable and uniform Zn deposition and improved cycling performance. These findings provide direct nanoscale insights into additive‐regulated Zn deposition and highlight Mn^2+^ ion‐mediated interfacial modulation as an effective strategy for controlling Zn morphology and enhancing AZIB durability.

## 2 Methods

Zinc electrodeposition was investigated by in situ EC‐LPTEM using a JEOL ARM‐200F NeoARM double aberration‐corrected transmission electron microscope equipped with a liquid‐flow electrochemistry holder (Stream Infinity, DENSsolutions) and corresponding flow‐cell chips (Stream Infinity Nano‐Cell, DENSsolutions).

**FIGURE 1:**
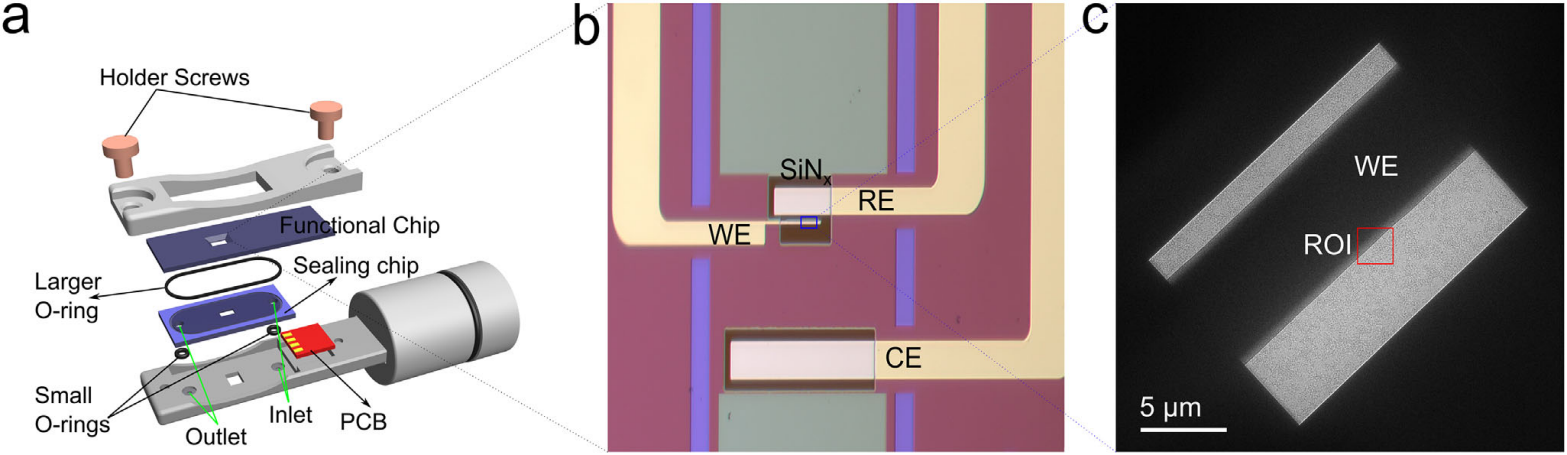
Schematics of the EC‐LPTEM holder and the functional chip for in situ electrochemical experiments. (a) Holder assembly. (b) Optical micrograph of the functional liquid cell chip, highlighting the SiN_x_ electron‐transparent membrane and the patterned Pt WE, CE, and RE. (c) Low‐magnification TEM image showing the observable region of the Pt WE and the relative position of the ROI selected for subsequent in situ observations.

For each in situ experiment, after assembling and tightening of the holder screws, the liquid inlet and outlet channels, as well as the electron‐transparent SiN_x_ membranes of the functional and sealing chips enclosing the liquid layer, were hermetically sealed by elastomeric O‐rings. Simultaneously, the functional chip established electrical contact with the printed circuit board (PCB). The electrolyte was introduced through the inlet of the sealing chip, where it flowed across the patterned electrodes on the functional chip. The solution subsequently exited through the outlet channel, forming a continuous flow path. The electrolyte flow was regulated by a syringe pump, while the liquid cell effectively isolated the aqueous environment from the high‐vacuum TEM column (Figure 1).

Platinum working (WE), counter (CE), and reference (RE) electrodes were patterned on the functional chip (Figure 1b). Zn electrodeposition on the WE was directly visualized through the electron‐transparent SiN_x_ membrane. The region of interest (ROI) was selected within the observable electrode area, as indicated in Figure 1c. Additional details of the EC‐LPTEM configuration are provided in the Supporting Information.

Zinc electrodeposition was investigated in two electrolytes: (i) 0.1 M ZnSO_4_ containing 20 mM MnSO_4_ (ZnSO_4_ + MnSO_4_ electrolyte) and (ii) 0.1 M ZnSO_4_ without additive (base ZnSO_4_ electrolyte). All experiments were conducted independently under identical electrochemical and imaging conditions to ensure comparability.

For each experiment, before applying an electrochemical potential, the electrolyte was continuously flowed through the liquid cell to remove bubbles and minimize beam‐induced radiolysis effects. The electron beam was maintained under the same imaging conditions used for the in situ observations for 20 min, during which no beam‐induced Zn deposition was detected.

To remove any possible beam‐induced Zn nucleation or residual deposits, a potential of +0.5 V was applied for 10 s prior to initiating Zn electrodeposition at a negative potential. No Zn deposition was detected at the onset of any experiment. Further details of the in situ experimental procedure are provided in the Supporting Information.

## 3 Results and Discussion

**FIGURE 2:**
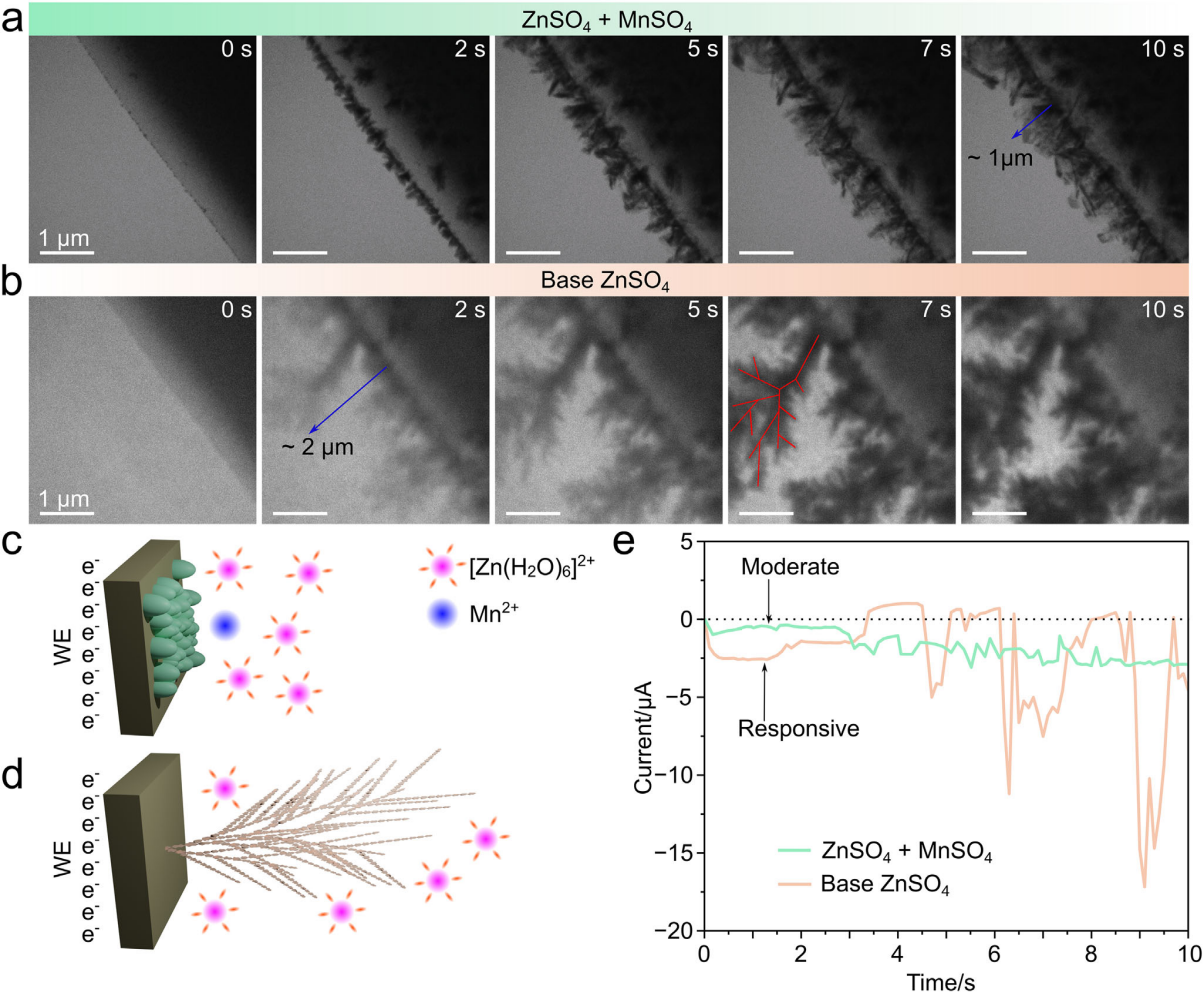
Time‐resolved TEM images of Zn electrodeposition under a −1.3 V applied potential and corresponding current–time responses. (a) Uniform Zn growth in ZnSO_4_ + MnSO_4_ electrolyte. (b) Heterogeneous and dendritic growth in base ZnSO_4_ electrolyte. (c, d) Schematic comparison of deposition behaviors. (e) Current–time profiles under the applied potential.

Zn electrodeposition imaging under a constant potential of −1.3 V for 10 s in the two electrolytes is shown in Figure 2. Clear differences in the nucleation behavior emerge at the early deposition stage (∼2 s). In the ZnSO_4_ + MnSO_4_ electrolyte (Figure 2a), nucleation sites are uniformly distributed across the WE surface, with a minimal size variation within the ROI.

In contrast, in the base ZnSO_4_ electrolyte without additive (Figure 2b), nucleation heterogeneity is immediately observed. Some nuclei rapidly evolve into dendritic structures, whereas others remain comparatively small, creating pronounced size disparity. Early‐stage dendrites can extend laterally beyond 2 µm. Although their thickness remains limited, the deposits preferentially elongate away from the electrode surface, indicating anisotropic growth.

During continuing polarization, the differences become increasingly pronounced. In the ZnSO_4_ + MnSO_4_ electrolyte, Zn deposition proceeds in a controlled, spatially uniform manner. The advancing deposition front remains nearly parallel to the electrode surface, reflecting homogeneous growth across the electrode–electrolyte interface. The deposition length increases gradually over time, reaching an average of ∼1 µm at 10 s, with a limited variation across electrode regions.

By contrast, deposition in the base ZnSO_4_ electrolyte proceeds in a highly heterogeneous manner. Rapidly emerging tree‐like structures exhibit markedly different growth rates. Existing dendrites thicken and branch, while new protrusions extend from pre‐formed structures, producing an irregular deposition front with pronounced localized growth. These observations indicate a morphologically unstable deposition process in the absence of Mn^2+^ ions.

Figure 2c,d schematically illustrates these distinct deposition behaviors. In the ZnSO_4_ + MnSO_4_ electrolyte, Zn forms a relatively uniform layer along the electrode surface, whereas in the base ZnSO_4_ electrolyte the deposits develop heterogeneous dendritic morphologies with significant spatial growth disparity.

The differences in plating dynamics are also reflected in the corresponding current–time profiles (Figure 2e). In the ZnSO_4_ + MnSO_4_ electrolyte, the current response during the initial nucleation stage is relatively moderate and subsequently evolves smoothly during continuing plating. In comparison, the base ZnSO_4_ electrolyte exhibits a more pronounced current response during nucleation, followed by noticeable fluctuations during deposition, consistent with the heterogeneous growth behavior observed in the in situ TEM images. Parasitic reactions, particularly the hydrogen evolution reaction (HER), may occur under the present experimental conditions and contribute to the observed current fluctuations [50, 51, 52]. Previous studies have suggested that Mn^2+^ additives can regulate Zn interfacial chemistry and mitigate parasitic reactions associated with HER in aqueous Zn systems [38, 53, 54]. However, whether, or to what extent, Mn^2+^ ions directly inhibit HER cannot be conclusively determined from the present EC‐LPTEM observations.

The significantly reduced growth heterogeneity and suppressed dendritic elongation observed in the Mn^2+^‐containing electrolyte suggest that Mn^2+^ ions mitigate localized growth instabilities while maintaining continuous Zn plating, thereby promoting more homogeneous electrodeposition under identical polarization conditions.

**FIGURE 3:**
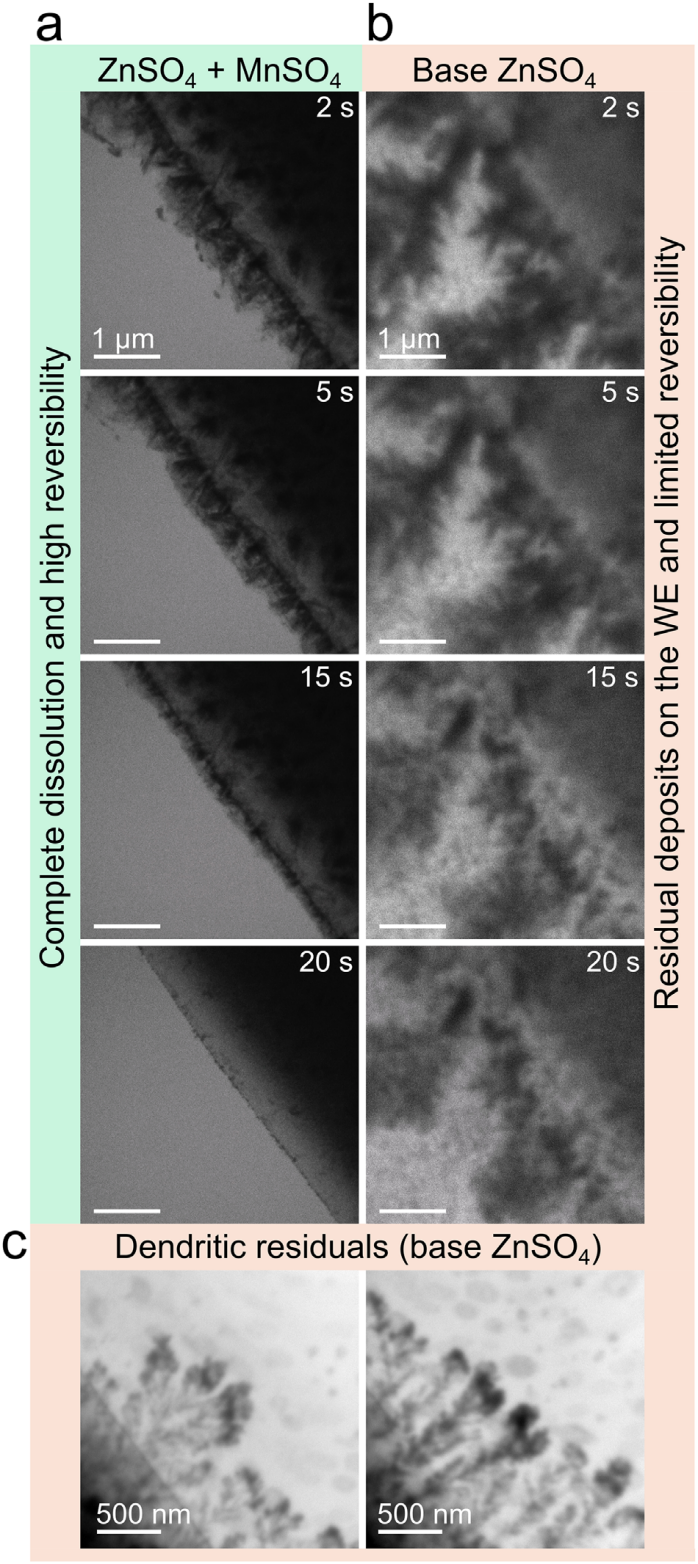
Time‐resolved TEM imaging of Zn deposit dissolution and residual dendritic structures in the base ZnSO_4_ electrolyte. (a) Deposits formed in the ZnSO_4_ + MnSO_4_ electrolyte dissolve gradually and nearly completely. (b) Incomplete dissolution in the base ZnSO_4_ electrolyte leaves substantial residual deposits on the WE. (c) Representative TEM images of residual deposits in the base ZnSO_4_ electrolyte showing retained dendritic morphology.

After removal of the applied negative potential, the potential of the WE returned to 0 V, and the electrodeposits underwent dissolution. The stripping behavior reveals clear differences in reversibility between the two electrolytes (Figure 3). Deposits formed in the ZnSO_4_ + MnSO_4_ electrolyte dissolve gradually and nearly completely within 20 s. In contrast, deposits formed in the base ZnSO_4_ electrolyte exhibit incomplete dissolution over the same period, with substantial residual material remaining attached to the electrode surface. Semi‐quantitative analysis of the residual dark‐contrast regions during stripping is provided in Figure S1. Some of the residual deposits in the base ZnSO_4_ electrolyte retain dendritic morphologies, as shown in Figure 3c.

Additional in situ experiments conducted under identical electrochemical conditions (Figures S2 and S3) further confirm the above observations. Overall, the distinct behaviors observed in situ indicate that the presence of Mn^2+^ ions regulates the interfacial deposition process, leading to more uniform Zn plating and improved reversibility under identical polarization conditions.

Previous in situ EC‐LPTEM studies investigated Zn plating/stripping in a 20 mM ZnSO_4_ + 5 mM MnSO_4_ electrolyte and reported Mn(OH)_2_ spherical particles near the working electrode, proposed to mechanically regulate local Zn^2+^ transport and thereby suppress dendritic Zn growth [47]. In the present work, the higher electrolyte concentration enabled more pronounced Zn deposition and interfacial evolution during plating and stripping to be captured. To further examine the morphology and chemical composition of the deposits formed in the ZnSO_4_ + MnSO_4_ electrolyte, post‐deposition TEM characterization was performed after removing the electrolyte from the liquid cell by compressed air flow. This reduced electron‐beam scattering from the liquid layer, improving imaging and diffraction resolution for morphology observation, SAED, and STEM‐EDS elemental mapping.

**FIGURE 4:**
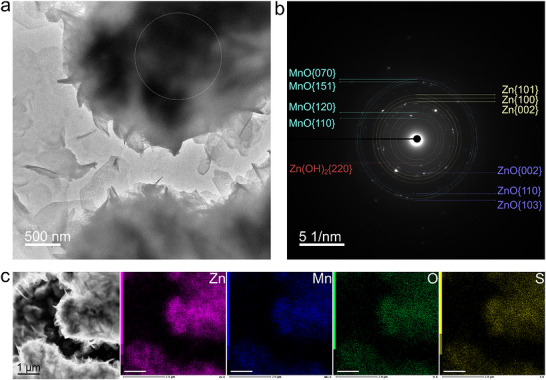
TEM, SAED, and STEM‐EDS characterizations of the deposits formed in the ZnSO_4_ + MnSO_4_ electrolyte. (a) TEM image showing bulky deposition near the WE and thin, flake‐like structures at the edges. (b) SAED pattern acquired from the region indicated in (a). (c) STEM‐ADF image and STEM‐EDS elemental mapping of Zn, Mn, O, and S corresponding to the close region shown in (a).

TEM and STEM‐ADF images (Figure 4) indicate that the Mn^2+^ additive exerts limited influence on the overall deposit morphology, particularly at the outer edges, where thin flake‐like structures are observed, similar to those formed in the base ZnSO_4_ electrolyte (Figure S4) [55]. Near the WE surface, the deposits appear relatively thick and bulky.

Additional structural information was obtained from the SAED patterns collected from regions adjacent to the WE (Figure 4a, white circle), as shown in Figure 4b. The deposits are predominantly composed of crystalline Zn, with strong diffraction spots corresponding to the Zn{002} and Zn{101} plane families (PDF#00‐004‐0831). Additional diffraction features associated with ZnO (PDF#00‐005‐0664) are also observed, while weaker spots attributable to Zn(OH)_2_ (PDF#00‐020‐1437) and MnO (PDF#00‐004‐0326) are detectable. Additional TEM imaging and corresponding SAED analysis are provided in Figure S5. Diffraction patterns collected from multiple interfacial regions consistently reveal the presence of Zn, ZnO, Zn(OH)_2_, and MnO.

The formation of Zn(OH)_2_ and ZnO in AZIBs has been widely reported and is generally attributed to parasitic reactions associated with the HER, in which Zn^2+^ combines with OH^−^ to form Zn(OH)_2_ that can subsequently dehydrate into ZnO [52, 56, 57]. The presence of MnO may arise from related interfacial reactions involving Mn^2+^ species under locally alkaline conditions generated during the deposition process. In analogy to Zn^2+^ chemistry, Mn^2+^ may also combine with OH^−^ to form Mn(OH)_2_, which could subsequently dehydrate to MnO. In addition to contributing to interfacial deposit formation, Mn^2+^ ions may modulate local interfacial chemistry through pH‐dependent hydroxide/oxide equilibria, thereby influencing the morphology and reversibility of the Zn–Mn–O–S deposits [54, 58, 59]. However, direct measurement of local pH evolution was beyond the capability of the present EC‐LPTEM experiments. Notably, no diffraction features corresponding to metallic Mn were observed, suggesting that Mn^2+^ ions do not undergo significant electrochemical reduction under the conditions investigated.

STEM‐EDS elemental mapping further confirms that Zn, Mn, O, and S are spatially correlated with the deposited features near the electrode (Figure 4c), while the corresponding STEM‐EDS sum spectrum is shown in Figure S6. The S signal is associated with the formation of commonly reported irreversible byproducts in ZnSO_4_‐based AZIBs, such as zinc hydroxysulfates (Zn_4_(OH)_6_SO_4_·xH_2_O, ZHS) [38, 60, 61, 62, 63, 64]. Previous studies suggest that Mn^2+^ ions can interact with ZHS species and thereby facilitate a more reversible deposition–dissolution process [41, 42, 65], consistent with the in situ observations, where the presence of Mn^2+^ ions leads to more uniform Zn plating and improved reversibility. The absence of Mn‐rich segregated phases indicates that Mn is incorporated within the interfacial deposits rather than forming separate domains. Together, these results indicate that Mn^2+^ ions modify Zn electrodeposition by influencing interfacial phase evolution and promoting the formation of mixed Zn–Mn–O‐S species, which are more chemically labile and contribute to uniform plating and enhanced reversibility compared with deposits formed in the additive‐free electrolyte.

The in situ TEM observations demonstrate that the presence of Mn^2+^ ions promotes more uniform Zn nucleation, controlled growth, and improved reversibility during the deposition–dissolution process. To evaluate whether these nanoscale behaviors translate into macroscopic electrochemical performance, the ex situ electrochemical measurements were performed using Zn‖Zn symmetric cells and V_2_O_5_‖Zn full cells [66]. For these measurements, more concentrated electrolytes (3 M ZnSO_4_ with 0.6 M MnSO_4_ additive and 3 M ZnSO_4_ without additive) were employed, a concentration commonly used in practical AZIB studies. Although higher in concentration than that used for in situ TEM measurements due to experimental constraints, the comparison between electrolytes with and without Mn^2+^ ions is well justified and enables a systematic evaluation of the additive's influence on electrochemical performance and post‐cycling Zn deposition morphology.

**FIGURE 5:**
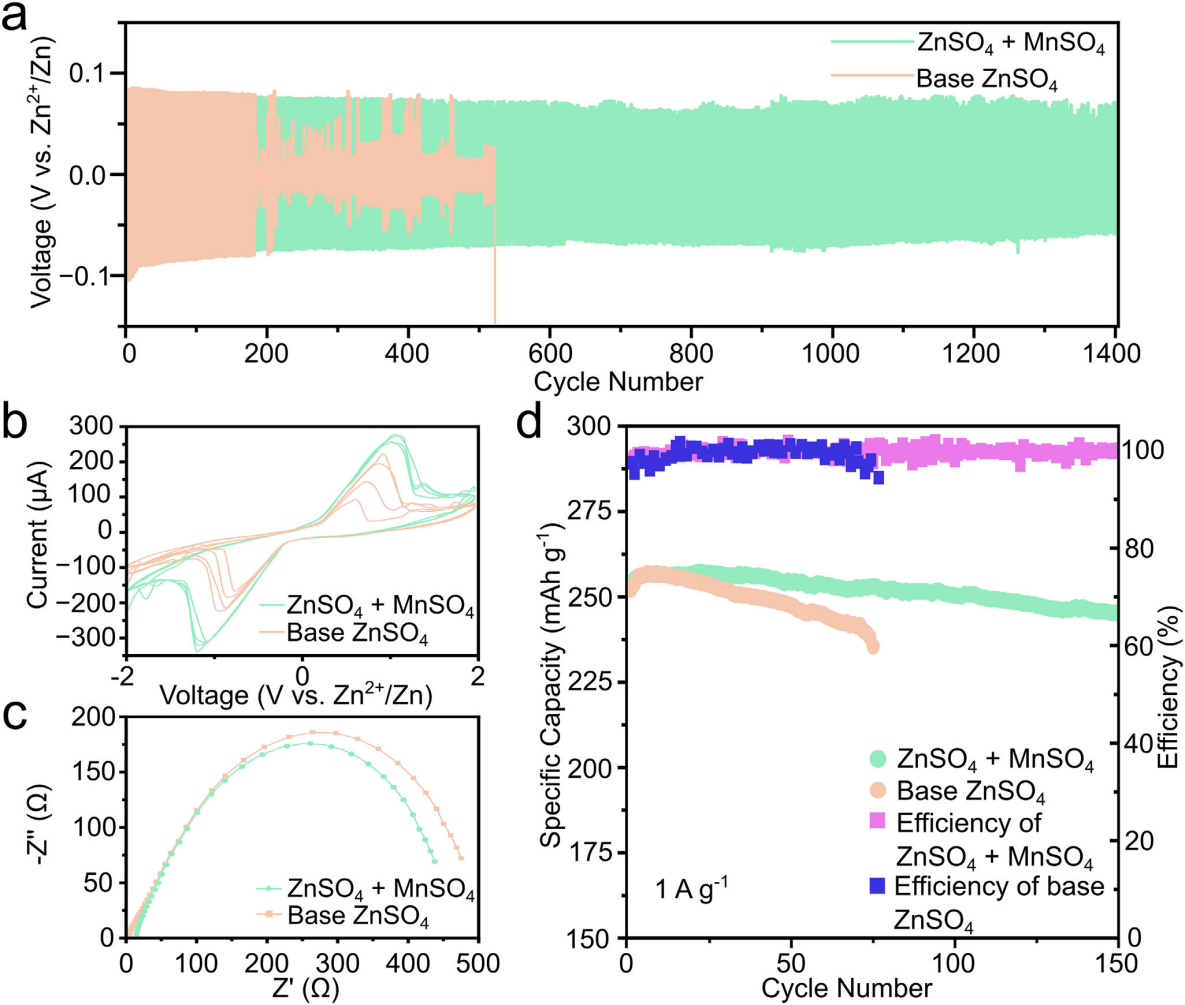
Electrochemical performance of cells using the ZnSO_4_ + MnSO_4_ electrolyte and the base ZnSO_4_ electrolyte. (a) Voltage profiles of symmetric Zn‖Zn cells during galvanostatic cycling. (b) CV curves in the two electrolytes. (c) Nyquist plots obtained from EIS measurements of Zn‖Zn symmetric cells. (d) Cycling performance of V_2_O_5_‖Zn full cells.

Figure 5 summarizes the electrochemical performance of CR2032 coin cells configured for Zn‖Zn symmetric and V_2_O_5_‖Zn full cells using the two electrolytes. Galvanostatic charge–discharge (GCD) cycling of Zn‖Zn symmetric cells (Figure 5a) shows that the cell using the ZnSO_4_ + MnSO_4_ electrolyte maintains relatively stable and reproducible plating/stripping voltage profiles over more than 1400 cycles. By contrast, the symmetric cell using the base ZnSO_4_ electrolyte exhibits similar voltage responses only during the initial ∼180 cycles, after which the voltage polarization rapidly decreases. This behavior may be associated with internal soft short‐circuit events arising from uneven Zn deposition [67, 68, 69]. The voltage subsequently recovers after several cycles, possibly due to detachment or dissolution of protruding deposits. During later cycles, the cell displays pronounced voltage fluctuations and eventually fails after 520 cycles.

Cyclic voltammetry (CV) measurements further highlight differences in the electrochemical stability of the two electrolytes (Figure 5b). In the ZnSO_4_ + MnSO_4_ electrolyte, successive scans exhibit nearly overlapping current–voltage curves, indicating stable and reproducible Zn plating/stripping behavior. In contrast, the CV curves recorded in the base ZnSO_4_ electrolyte gradually diverge, reflecting less stable interfacial reactions. Moreover, the ZnSO_4_ + MnSO_4_ electrolyte exhibits a slightly larger integrated peak area, indicating enhanced electrochemical activity during the plating/stripping process.

Electrochemical impedance spectroscopy (EIS) of the symmetric cells (Figure 5c) reveals a depressed semicircle in the high‐to‐medium frequency region, characteristic of charge‐transfer‐controlled interfacial processes. Equivalent‐circuit fitting (Figure S7) was performed to quantitatively extract the solution resistance (R_s_) and charge‐transfer resistance (R_ct_). The ZnSO_4_ + MnSO_4_ electrolyte exhibits a slightly higher R_s_. However, the most notable change is the decrease in R_ct_ from 457.8 Ω for the base ZnSO_4_ electrolyte to 362.3 Ω for the ZnSO_4_ + MnSO_4_ electrolyte, indicating a more favorable Zn^2+^ deposition/stripping interface and facilitated Zn deposition/stripping kinetics in the presence of Mn^2+^ ions.

The influence of Mn^2+^ is also apparent in V_2_O_5_‖Zn full cells. At 1 A g^−^^1^, both cells initially deliver capacities of approximately 260 mAh g^−^^1^ with nearly 100% Coulombic efficiency (Figure 5d). However, the cell using the base ZnSO_4_ electrolyte undergoes rapid capacity decay, failing after 75 cycles, whereas the cell with the ZnSO_4_ + MnSO_4_ electrolyte maintains stable cycling for nearly 150 cycles with a gradually declining capacity.

The surface morphologies of Zn‖Zn symmetric cell electrodes after long‐term GCD cycling were examined by SEM (Figure S8). Deposits formed in the ZnSO_4_ + MnSO_4_ electrolyte exhibit a relatively smooth surface, whereas those formed in the base ZnSO_4_ electrolyte show widely distributed spike‐like structures with sharp tips extending outward, predominantly along directions close to the surface normal. Such anisotropic protrusions may increase the likelihood of soft short‐circuit events, thereby contributing to the pronounced voltage fluctuations and earlier failure observed in Figure 5a.

**FIGURE 6:**
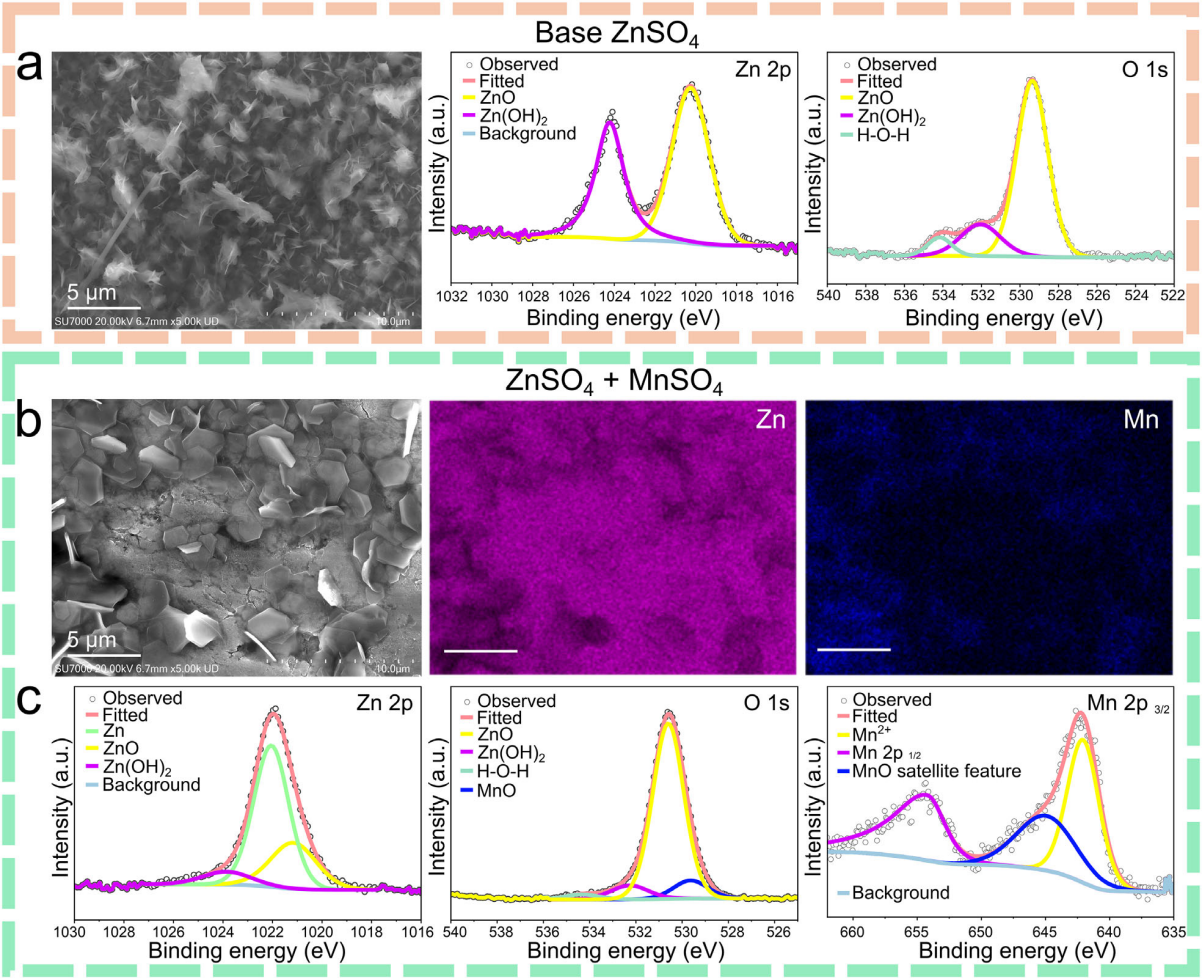
SEM, SEM‐EDS, and XPS analyses of Zn deposits after initial cycling (10 GCD cycles). (a) Base ZnSO_4_ electrolyte: spike‐like deposits with sharp tips; presence of ZnO and Zn(OH)_2_. (b) ZnSO_4_ + MnSO_4_ electrolyte: no pronounced protrusions; Zn and Mn distributions align with deposits features. (c) ZnSO_4_ + MnSO_4_ electrolyte: dominant Zn signals, reduced adsorbed water, and Mn primarily in the Mn^2+^ state.

SEM‐EDS and X‐ray photoelectron spectroscopy (XPS) analyses of electrode surfaces after long‐term cycling show no detectable Mn signals, likely due to coverage by thick deposits formed during extended cycling. To further probe the role of Mn^2+^ ions during the early stages of electrodeposition, SEM and XPS analyses were conducted on Zn‖Zn symmetric cells after 10 GCD cycles (Figure 6).

In the base ZnSO_4_ electrolyte (Figure 6a), spike‐like deposits with sharp outward‐pointing tips are widely distributed across the electrode surface. The Zn 2p_3/2_ XPS spectrum exhibits two distinguishable components at ∼1020.4 and ∼1024.2 eV, which can be attributed to Zn^2+^ species, such as ZnO and Zn(OH)_2_ [70, 71]. The O 1s spectrum exhibits a component at ∼534.1 eV, commonly attributed to adsorbed water or hydroxyl‐containing species [72].

In the ZnSO_4_ + MnSO_4_ electrolyte (Figure 6b), the deposits show no pronounced protrusions. SEM‐EDS elemental mapping shows that Zn and Mn are spatially correlated with the deposited features (O and S distributions are shown in Figure S9). The Zn 2p_3/2_ spectrum is dominated by a peak at ∼1021.8 eV, Zn deposition remains the primary process during the initial cycling stage [73]. The O 1s spectrum exhibits a reduced contribution from the high‐binding‐energy component associated with adsorbed water, suggesting a modified interfacial environment in the presence of Mn^2+^ [41].

The Mn 2p spectrum indicates that Mn is predominantly present in the Mn^2+^ state, without undergoing significant redox transformation. Weak satellite features may be associated with Mn–O species, consistent with the SAED observations following the in situ measurements [74].

Overall, results suggest that Mn^2+^ ions participate in the interfacial processes during early‐stage deposition without forming distinct segregated phases, leading to a modified interfacial chemistry that correlates with the more uniform morphology and improved reversibility observed in situ.

## 4 Conclusions

In summary, this work employs in situ EC‐LPTEM to directly visualize, in real time, the role of Mn^2+^ ions in regulating Zn electrodeposition and dissolution at the nanoscale. The presence of Mn^2+^ ions is shown to suppress growth heterogeneity and dendritic elongation during plating, while promoting more complete and reversible stripping. Although the electrolyte concentrations used in the in situ EC‐LPTEM experiments and complementary ex situ electrochemical measurements differed substantially due to experimental constraints, the primary objective was to evaluate the relative influence of Mn^2+^ ions on Zn deposition behavior rather than directly correlate absolute electrochemical behavior between the two systems. Complementary ex situ electrochemical measurements further demonstrate enhanced Zn anode stability and cycling performance, consistent with the deposition behavior observed in situ.

By capturing additive‐regulated deposition dynamics with nanoscale resolution, this study provides direct experimental evidence of how dissolved metal salt additives modulate interfacial processes in aqueous electrolytes. The results indicate that Mn^2+^ ions influence Zn deposition without undergoing significant redox transformation, while modulating interfacial processes during plating and stripping.

These findings establish a mechanistic link between electrolyte composition and Zn deposition behavior, offering design principles for advanced electrolytes that enable uniform plating and improved reversibility. More broadly, this work highlights the utility of in situ EC‐LPTEM for uncovering dynamic interfacial processes and guiding the rational development of durable aqueous zinc‐ion batteries.

## Author Contributions

**Joseph F.S. Fernando**: software, supervision, validation. **Kudachchige Asanga G. de Alwis**: conceptualization, methodology. **Dasun P. W. Guruge**: methodology. **Dmitri V. Golberg**: supervision, resources, funding acquisition, writing – review and editing, project administration. **Zhenhuan Chen**: conceptualization, investigation, writing – original draft, visualization, methodology, software, data curation, formal analysis, validation. **Kaveendra V. Maduwantha**: software. **Chao Zhang**: supervision, writing – review and editing. **Konstantin L. Firestein**: conceptualization, supervision.

## Conflicts of Interest

The authors declare no conflicts of interest.

## Supporting information




**Supporting File 1**: advs76489‐sup‐0001‐SuppMat.docx.

**Supporting File 2**: advs76489‐sup‐0002‐VideoS1.mp4.

**Supporting File 3**: advs76489‐sup‐0003‐VideoS2.mp4.

**Supporting File 4**: advs76489‐sup‐0004‐VideoS3.mp4.

**Supporting File 5**: advs76489‐sup‐0005‐VideoS3.mp4.

## Data Availability

The data that support the findings of this study are available from the corresponding author upon reasonable request.
