# Supplementary material for: Mn2+ Ion‐Mediated Zn Dendrite Suppression and Reversible Stripping in Aqueous Zn‐Ion Batteries as Revealed by In Situ Liquid‐Cell TEM
Source: Adv Sci (Weinh). 2026 Jul 16;13(55):e76489. doi: 10.1002/advs.76489 (PMC13373903; doi:10.1002/advs.76489)
Supplement: Supplementary file 1 [file ADVS-13-e76489-s001.docx]

**Mn^2+^ ion-mediated Zn dendrite suppression and reversible stripping in aqueous Zn-ion batteries as revealed by in situ liquid-cell TEM**

*Zhenhuan Chen,* *Dasun P. W. Guruge, Kudachchige Asanga G. de Alwis**,* *Kaveendra V. Maduwantha, Konstantin L. Firestein,* *Joseph F.S. Fernando, Chao Zhang***, Dmitri V. Golberg**

Corresponding Authors:

Chao Zhang − Central Analytical Research Facility and Centre for Materials Science, Queensland University of Technology (QUT), 2 George St, Brisbane, Queensland, 4000, Australia; https://orcid.org/0000-0001-5309-8484

E-mail: [ch.zhang@qut.edu.au](mailto:ch.zhang@qut.edu.au)

Dmitri V. Golberg − School of Chemistry and Physics and Centre for Materials Science, Queensland University of Technology (QUT), 2 George St, Brisbane, Queensland, 4000, Australia; https://orcid.org/0000-0003-2298-6539

E-mail: [dmitry.golberg@qut.edu.au](mailto:dmitry.golberg@qut.edu.au)

Authors:

Zhenhuan Chen − https://orcid.org/0009-0004-8880-929X

Dasun P. W. Guruge − https://orcid.org/0009-0000-2161-4595

Kudachchige Asanga G. de Alwis − https://orcid.org/0009-0008-8008-1125

Kaveendra V. Maduwantha − https://orcid.org/0000-0002-1049-7829

School of Chemistry and Physics and Centre for Materials Science, Queensland University of Technology (QUT), 2 George St, Brisbane, Queensland, 4000, Australia.

Konstantin L. Firestein − Central Analytical Research Facility and Centre for Materials Science, Queensland University of Technology (QUT), 2 George St, Brisbane, Queensland, 4000, Australia; https://orcid.org/0000-0002-0928-6477

Joseph F.S. Fernando − Centre for Microscopy and Microanalysis, The University of Queensland, St Lucia, Queensland 4072, Australia, School of Chemistry and Physics and Centre for Materials Science, Queensland University of Technology (QUT), 2 George St, Brisbane, Queensland, 4000, Australia; https://orcid.org/0000-0003-0129-359X

**Supporting Information**

During the stripping process, the dark-contrast regions in the time-resolved TEM images correspond to the remaining deposits on the working electrode. To semi-quantitatively compare the dissolution behavior in the two electrolytes, the dark-contrast pixels in each frame were counted and normalized to those in the initial stripping frame. The resulting evolution profiles of the residual deposits are shown in Figure S1. The analysis reveals that only ~3.5% of the residual deposits remain on the electrode within 20 s in the ZnSO_4_ + MnSO_4_ electrolyte, whereas ~71% of the deposits remain in the base ZnSO_4_ electrolyte over the same stripping period under otherwise identical experimental conditions.

**Figure S1.** Evolution of the residual deposit fraction during the stripping process in the two electrolytes.

Additional in situ observations of Zn electrodeposition under identical electrochemical and imaging conditions are shown in Figure S2.

The results are consistent with those presented in the main text. During plating, the ZnSO_4_ + MnSO_4_ electrolyte exhibits reduced growth heterogeneity and suppressed dendritic elongation, indicating more homogeneous electrodeposition. In contrast, the base ZnSO_4_ electrolyte shows pronounced morphological instability.

During stripping, deposits formed in the ZnSO_4_ + MnSO_4_ electrolyte dissolve gradually and nearly completely, whereas those formed in the base ZnSO_4_ electrolyte exhibit incomplete dissolution over the same period.

The corresponding current–time profiles (Figure S3) further support these observations. The ZnSO_4_ + MnSO_4_ electrolyte shows a moderate current response during nucleation followed by smooth evolution during growth, while the base ZnSO_4_ electrolyte exhibits a higher initial current and noticeable fluctuations during deposition.

Overall, these results confirm the reproducibility of the in situ observations and demonstrate that Mn^2+^ ions regulate interfacial Zn deposition, leading to more uniform plating and improved reversibility under identical polarization conditions.

**Figure S2.** Time-resolved TEM images of Zn electrodeposition under a −1.3 V applied potential and the subsequent stripping at 0 V under identical conditions. (a) Uniform Zn nucleation and growth in the ZnSO_4_ + MnSO_4_ electrolyte. (b) Heterogeneous nucleation and dendritic growth in the base ZnSO_4_ electrolyte. (c) Deposits formed in the ZnSO_4_ + MnSO_4_ electrolyte dissolve gradually and nearly completely during stripping. (d) Incomplete dissolution in the base ZnSO_4_ electrolyte, leaving substantial residual deposits.

**Figure S3.** Corresponding current–time profiles for the additional in situ electrodeposition experiments. The ZnSO_4_ + MnSO_4_ electrolyte exhibits a moderate initial current response followed by smooth evolution during deposition, whereas the base ZnSO_4_ electrolyte shows a higher initial current and noticeable fluctuations, indicative of unstable growth.

The deposits formed in the base ZnSO_4_ electrolyte exhibit thin, flake-like features at the tip regions, while the regions adjacent to the working electrode (WE) appear relatively thick and bulky. Similar morphological features are also observed in the ZnSO_4_ + MnSO_4_ electrolyte.

**Figure S4.** TEM image of deposits formed in the base ZnSO_4_ electrolyte. Thin, flake-like features are observed at the deposit tips, similar to those formed in the ZnSO_4_ + MnSO_4_ electrolyte.

Additional TEM imaging and corresponding SAED analysis were performed on other representative regions of deposits formed in the ZnSO_4_ + MnSO_4_ electrolyte. TEM images show thin, flake-like structures at the deposit edges. SAED patterns collected from regions adjacent to the WE reveal the coexistence of Zn (PDF#00-004-0831), ZnO (PDF#00-005-0664), Zn(OH)_2_ (PDF#00-020-1435), and MnO (PDF#00-004-0326), consistent with the analysis presented in the main text.

**Figure S5.** TEM and SAED characterizations of deposits formed in the ZnSO_4_ + MnSO_4_ electrolyte. (a,c) TEM images of representative regions showing thin, flake-like structures at the deposit edges. (b,d) SAED patterns acquired from the regions indicated by white circles in (a) and (c), respectively.

The STEM-EDS sum spectrum of the STEM-EDS elemental mapping in Figure 4c is presented in Figure S6, with the characteristic Zn, O, S, and Mn peaks labelled. The elemental maps were generated from the STEM-EDS spectrum imaging dataset using JEOL Analysis Station by integrating the characteristic X-ray signals of the selected elements at each scan pixel. Background correction was performed by the analysis software to obtain the corresponding elemental distribution maps.

**Figure S6.** STEM-EDS sum spectrum with characteristic Zn, O, S, and Mn peaks labelled.

Equivalent-circuit fitting has been performed using Gamry Echem Analyst to quantitatively extract the solution resistance (R_s_) and charge-transfer resistance (R_ct_) values (Figure S7).

**Figure S7.** Equivalent circuit model used for fitting the EIS spectra of the Zn||Zn symmetric cells.

The fitting results show that the ZnSO_4_ + MnSO_4_ electrolyte exhibits an R_s_ of 17.5 ± 0.2 Ω and an R_ct_ of 362.3 ± 15.5 Ω, whereas the ZnSO_4_ electrolyte exhibits an R_s_ of 1.7 ± 0.2 Ω and an R_ct_ of 457.8 ± 26.4 Ω.

The fitting results confirm that the ZnSO_4_ + MnSO_4_ electrolyte exhibits a slightly higher R_s_ but lower R_ct_ compared with the base ZnSO_4_ electrolyte, indicating improved Zn deposition/stripping kinetics in the presence of Mn^2+^ ions.

The surface morphologies of Zn‖Zn symmetric cell electrodes after long-term GCD cycling were examined by SEM (Figure S8).

Here, “long-term” refers to electrodes subjected to extended cycling (up to ~520 cycles for the base ZnSO_4_ electrolyte and over 1400 cycles for the ZnSO_4_ + MnSO_4_ electrolyte, as shown in Figure 5a).

To ensure a systematic comparison, all images were acquired at identical magnification using the same secondary electron detector.

After prolonged cycling, deposits formed in the ZnSO_4_ + MnSO_4_ electrolyte exhibit relatively smooth surfaces without evident sharp protrusions. In contrast, those formed in the base ZnSO_4_ electrolyte show widely distributed spike-like structures with pronounced, sharp tips extending outward, predominantly along directions close to the surface normal. Such anisotropic, outward-growing features are characteristic of dendritic growth and may increase the likelihood of soft short-circuit events, thereby contributing to the pronounced voltage fluctuations and earlier failure observed in Figure 5a.

It should be noted that the in situ EC-LPTEM experiments and ex situ GCD measurements were performed under different electrochemical control modes. Therefore, the deposition/dissolution kinetics and interfacial reaction behavior observed during EC-LPTEM may differ quantitatively from those in practical battery cycling conditions. In particular, the potential-relaxation process during the EC-LPTEM stripping experiments may facilitate the dissolution of chemically labile interfacial deposits. Nevertheless, both the in situ and ex situ experiments consistently reveal that Mn^2+^ ions suppress heterogeneous Zn growth and improves electrode reversibility.

SEM-EDS and XPS analyses of the cycled electrodes show only weak Mn signals. This may be attributed to the burial of Mn-containing species beneath relatively thick deposits after prolonged cycling. Despite the limited detectable Mn signal, the deposits formed in the two electrolytes exhibit clearly distinct surface morphologies.

**Figure S8.** SEM images of Zn electrodes from Zn‖Zn symmetric coin cells after long-term cycling in ZnSO_4_ + MnSO_4_ and base ZnSO_4_ electrolytes. (a,b) ZnSO_4_ + MnSO_4_ electrolyte: relatively smooth surfaces without evident sharp or protruding features. (c,d) Base ZnSO_4_ electrolyte: widely distributed spike-like deposits with pronounced, sharp tips extending outward from the surface.

SEM-EDS elemental mapping of deposits formed in the ZnSO_4_ + MnSO_4_ electrolyte after initial cycling (first 10 cycles) is shown in Figure S9. Zn, Mn, O, and S signals are spatially correlated with the deposited features.

**Figure S9.** SEM-EDS elemental mapping of deposits formed in the ZnSO₄ + MnSO₄ electrolyte after initial cycling (first 10 cycles), showing the spatial distribution of Zn, Mn, O, and S.

The corresponding SEM-EDS spectrum is presented in Figure S10, showing the relative intensities of Zn, O, S, and Mn. C signal is also detected, which is attributed to the glass microfiber separator used in the cell.

**Figure S10.** SEM-EDS spectrum corresponding to the elemental mapping in Figure S6.

**Experimental Methods**

In Situ Electrodeposition for EC-LPTEM

In situ electrodeposition was conducted using a liquid flow electrochemistry holder (Stream Infinity, DENSsolutions), coupled with SiN_x_ liquid-cell chips (Stream Infinity Nano-Cell, DENSsolutions).

The liquid layer thickness was maintained at ~200 nm upon tightening the holder screws, which compresses the elastomeric O-rings (Figure 1). Variations in liquid thickness between different assembly rounds, as well as pressure fluctuations between the membranes during experiments, can affect imaging resolution.

The electrolyte was introduced through the holder inlet and directed to the sealing chip inlet, from which it entered the functional chip and contacted the patterned electrodes. The solution then exited through the sealing chip outlet and reached the holder outlet, completing a continuous flow path.

The liquid-cell holder chassis was electrically grounded to ensure a stable negative potential applied to the working electrode. Electrochemical measurements were performed using a pseudo-reference electrode integrated into the liquid-cell configuration. In all experiments, the applied potential was maintained for a short duration (10 s), minimizing the influence of potential drift during in situ observations.

The grounding cable and the inlet/outlet tubing of the holder are indicated in Figure S11.

**Figure S11.** Experimental setup for the in situ EC-LPTEM. The liquid-cell holder chassis is electrically grounded. The electrolyte is introduced through the holder inlet, flows into the sealing chip and functional chip (Figure 1), and exits through the holder outlet to a waste container, forming a continuous flow path.

Solution flow was maintained at ~4 μL/min using a syringe pump, and the applied pressure was continuously monitored with a pressure sensor. The SiN_x_ membranes remain mechanically stable up to ~4500 mbar. The tubing connected to the pump, as marked in Figure S11, is also connected to a compressed air line. When the tubing is switched to the gas line, compressed air is introduced into the tubing system at a set pressure to remove the electrolyte from the liquid cell. The applied pressure for electrolyte removal is typically higher than that used for liquid flow, but it remains well below the membrane failure threshold. Removal of the electrolyte enables higher-resolution TEM imaging, as shown in Figure 4.

All electrochemical processes were observed through the SiN_x_ windows at an acceleration voltage of 200 kV using JEOL ARM-200F NeoARM double aberration-corrected transmission electron microscope.

Electron beam–induced effects, including radiolysis of the aqueous electrolyte and possible beam-driven nucleation, cannot be completely excluded in liquid-phase TEM experiments [1]. Radiolysis may generate reactive species (e.g., solvated electrons, H^+^, OH^-^), which can influence local electrochemical environments and potentially affect Zn deposition behavior [2]. Due to experimental limitations, accurate determination of the electron dose rate during in situ observations remains challenging. To ensure comparability and clear imaging conditions while minimizing possible electron beam damage, identical imaging conditions were maintained across all experiments. Specifically, a 150 μm condenser aperture and a 30 μm objective aperture were used, with a spot size of 2. Before inserting the CCD camera, the electron beam was spread to the same extent on the fluorescent screen. A beam shutter was then employed to deflect approximately 85% of the beam, thereby reducing beam-induced effects while maintaining sufficient image contrast.

For each in situ experiment, after introducing the electrolyte into the liquid cell, the solution was continuously flowed to remove bubbles and mitigate beam-induced radiolysis effects. The electron beam conditions were stabilized for 20 min prior to applying any electrochemical potential, during which no beam-induced Zn deposition was observed. For each experiment, before applying a negative potential to initiate Zn electrodeposition, a potential of +0.5 V was applied for 10 s to eliminate any possible beam-induced Zn nucleation or residual deposits on the electrode surface. No Zn deposition was observed at the onset of any experiment.

**Figure S12.** TEM images demonstrating the absence of beam-induced Zn deposition prior to electrochemical measurements. (a) Before electrolyte introduction. (b) After electrolyte introduction, with the electron beam being maintained for 20 min without applied potential; no Zn deposition is observed.

The use of liquid flow cells enables continuous electrolyte refreshment during imaging, which helps mitigate electron-beam-induced effects by removing radiolysis products and gas bubbles. Prior to applying any electrochemical potential, no beam-induced nucleation or deposition was observed, and no observable Zn deposition occurred during beam exposure in the absence of applied potential (Figure S12), suggesting that beam-induced deposition is negligible under the present conditions.

The EC-LPTEM experiments were performed under continuous electrolyte flow (~4 μL/min), with flow-rate fluctuations maintained below ~3% according to the flowmeter readings. Considering the relatively short experimental duration (~10 s), the influence of flow-rate variation during individual in situ observations is expected to be minimal. Apart from removing gas bubbles and radiolysis products, the flowing electrolyte in EC-LPTEM also helps maintain a fresh ion supply near the electrode surface and reduces localized concentration gradients. Since the ex situ coin-cell measurements were conducted under static electrolyte conditions, the mass transport conditions in EC-LPTEM may quantitatively differ from those in practical coin cells and could influence interfacial deposition/dissolution kinetics. Nevertheless, the in situ experiments were designed to comparatively evaluate the relative effects of Mn^2+^ ions on Zn deposition and dissolution behavior under controlled and reproducible conditions.

Electrolytes preparation

The electrolytes were prepared using zinc sulfate heptahydrate (ZnSO_4_·7H_2_O, Sigma-Aldrich, >99.0%) and manganese(II) sulfate monohydrate (MnSO_4_·H_2_O, Sigma-Aldrich, ≥99%) for the ZnSO_4_ + MnSO_4_ and base ZnSO_4_ electrolytes, used in both in the in situ experiments and ex situ electrochemical measurements.

It should be noted that electrolyte concentration can influence Zn^2+^ solvation/desolvation behavior, interfacial reaction kinetics, and interfacial deposit formation. Therefore, the dilute electrolyte environment used in EC-LPTEM may differ quantitatively from practical battery systems in deposition kinetics and interfacial layer growth [3, 4]. Nevertheless, the in situ observations qualitatively reveal that Mn^2+^ ions suppress growth heterogeneity and dendritic elongation during Zn plating while promoting more reversible Zn stripping. The electrolyte concentration used for the in situ EC-LPTEM experiments was substantially lower than that used in the ex situ electrochemical measurements due to the technical limitations of liquid-phase TEM. Conventional AZIB electrolyte concentrations (1–3 M) are too viscous for a stable flow in SiN_x_ liquid cells. With a liquid layer thickness of ~200 nm, 0.1 M ZnSO_4_ already produces clearly observable Zn electrodeposition, consistent with prior liquid-phase TEM studies using similarly low concentrations (e.g., 0.3 M [5], 0.1 M [6-8], and 0.02M [9, 10]). Higher concentrations would induce excessively rapid and aggressive deposition, increasing the risk of SiN_x_ membrane penetration and TEM vacuum failure.

Previous ex situ studies have reported a broad range of MnSO_4_/ZnSO_4_ concentration ratios (from 1:1 to 1:10) in Mn^2+^ ion-containing aqueous Zn systems [11-13]. Based on these previous reports and the stability requirements of EC-LPTEM experiments, 20 mM MnSO_4_ was selected as the additive concentration in combination with 0.1 M ZnSO_4_, corresponding to a moderate additive-to-electrolyte ratio suitable for stable in situ observation.

Coin-cell assembly

CR2032 coin cells were assembled for symmetric Zn‖Zn and V_2_O_5_||Zn full-cell tests. Stainless-steel cases, standard spacers, and springs were used, separated by glass microfiber filters. Electrolytes consisted of either 3 M ZnSO_4_ with 0.6 M MnSO_4_ additive or 3 M ZnSO_4_ without additive. Symmetric cells employed 0.25 mm zinc foil as both working and counter electrodes. Full cells employed 0.25 mm zinc foil as the anode and a V_2_O_5_-based nanostrand cathode with active material loading of ~3–5 mg/cm². All cells were sealed and allowed to equilibrate for 48 h before electrochemical testing.

Cathode active material and slurry preparation

V_2_O_5_ nanostrands were synthesized by adding 1.0 g commercial V_2_O_5_ powder to 15 mL of 2.0 M KCl aqueous solution and stirring at room temperature for 72 h. The mixture was filtered, vacuum-dried at 30 °C for 24 h, and ground into fine powder. The powder was mixed with Super P carbon black and PVDF binder (pre-dissolved in 6 wt% N-methyl-2-pyrrolidone, NMP) in a 7:2:1 mass ratio, and stirred for ~24 h to form a homogeneous slurry. The slurry was uniformly coated onto plasma-treated carbon fiber cloth current collectors and vacuum-dried at 50 °C for 24 h. Electrode discs were punched, weighed to determine active material loading, and stored in a dry environment prior to cell assembly [14].

Ex situ electrochemical characterization

Galvanostatic charge-discharge (GCD) measurements were performed using a LANHE CT2001 electrochemical workstation. Cyclic voltammetry (CV) was conducted with a Keithley 2450 SourceMeter, and electrochemical impedance spectroscopy (EIS) was measured using a Gamry Interface 1010E over a frequency range of 0.1 kHz to 1 MHz.

Although the cycling performance of the present V_2_O_5_||Zn full cell remains below that of some recently reported optimized AZIB systems, the primary focus of this work was to establish direct nanoscale visualization of Mn^2+^ ion-mediated Zn deposition regulation using in situ EC-LPTEM. The improved cycling stability observed in the Mn^2+^-containing electrolyte is consistent with the suppressed dendritic Zn growth and enhanced stripping reversibility directly observed during the in situ experiments.

Post-cycling SEM and XPS characterization

Post-cycling surface characterizations were performed on electrodes disassembled from Zn‖Zn symmetric cells (Figure S13), with all analyses conducted on the side facing the separator.

**Figure S13.** Electrodes disassembled from Zn‖Zn symmetric cells; surface characterizations were performed on the side adjacent to the separator.

SEM analysis

After GCD cycling, the electrode surface morphology was examined using a Hitachi SU7000 field-emission scanning electron microscope at 1.5 kV accelerating voltage and 6.0–6.6 mm working distance. All images were acquired at identical magnification using the same secondary electron detector to ensure systematic comparison. SEM-EDS was performed at 20 kV to map elemental distributions.

XPS analysis

XPS was conducted using a Kratos Axis Supra instrument with an Al Kα monochromatic X-ray source (h𝜈 = 1486.69 eV), emission current of 15 mA, and X-ray power of 225 W. Data analysis was carried out using CASAXPS software with Kratos relative sensitivity factors (RSFs). All binding energies were calibrated using the C 1s peak at 284.8 eV.

**Captured Videos**

Captured videos (.mp4) of zinc electrodeposition in the two electrolytes:

ZnSO_4_ + MnSO_4_ (Figure 2a, Figure 3a).

Base ZnSO_4_ (Figure 2b, Figure 3b).

ZnSO_4_ + MnSO_4_-SI (Figure S1a,c).

Base ZnSO_4_-SI (Figure S1b,d).

**References**

1	N. De Jonge and F. M. Ross, “Electron Microscopy of Specimens in Liquid,” *Nature Nanotechnology* 6 (2011): 695-704. <https://dx.doi.org/10.1038/NNANO.2011.161>

2	N. M. Schneider, M. M. Norton, B. J. Mendel, et al., “Electron–Water Interactions and Implications for Liquid Cell Electron Microscopy,” *The Journal of Physical Chemistry C* 118 (2014): 22373-22382. <https://dx.doi.org/10.1021/jp507400n>

3	J. Cao, D. Zhang, X. Zhang, et al., “Strategies of Regulating Zn^2+^ Solvation Structures for Dendrite-Free and Side Reaction-Suppressed Zinc-Ion Batteries,” *Energy & Environmental Science* 15 (2022): 499-528. <https://dx.doi.org/10.1039/D1EE03377H>

4	H. Wang, K. Wang, E. Jing, et al., “Strategies of Regulating Zn^2+^ Solvation Structures Toward Advanced Aqueous Zinc-Based Batteries,” *Energy Storage Materials* 70 (2024): 103451. <https://dx.doi.org/10.1016/j.ensm.2024.103451>

5	M. Li, L. Ran, and R. Knibbe, “Zn Electrodeposition by an In Situ Electrochemical Liquid Phase Transmission Electron Microscope,” *The Journal of Physical Chemistry Letters* 12 (2021): 913-918. <https://dx.doi.org/10.1021/acs.jpclett.0c03475>

6	Y. Sasaki, K. Yoshida, T. Kawasaki, et al., “In Situ Electron Microscopy Analysis of Electrochemical Zn Deposition onto an Electrode,” *Journal of Power Sources* 481 (2021): 228831. <https://dx.doi.org/10.1016/j.jpowsour.2020.228831>

7	J. Park, S. Dutta, H. Sun, et al., “Toward Quantitative Electrodeposition via In Situ Liquid Phase Transmission Electron Microscopy: Studying Electroplated Zinc Using Basic Image Processing and 4D STEM,” *Small Methods* 8 (2024): 2400081. <https://dx.doi.org/10.1002/smtd.202400081>

8	W. Dachraoui, R.-S. Kuhnel, C. Battaglia, and R. Erni, “In Situ Liquid-Phase Transmission Electron Microscopy Investigation of Anode–Electrolyte Interface Processes in Zn-Ion Batteries,” *ACS Nano* 19 (2025): 42916-42927. <https://dx.doi.org/10.1021/acsnano.5c16519>

9	H. Zeng, R. Huang, J. Lin, et al., “Shape-Controlled Zn Nanosheet Electrodeposition Revealed by In Situ Liquid-Phase Transmission Electron Microscopy,” *The Journal of Physical Chemistry C* 127 (2023): 22992-22999. <https://dx.doi.org/10.1021/acs.jpcc.3c06164>

10	Y. Huang, Q. Gu, Z. Guo, et al., “Unraveling Dynamical Behaviors of Zinc Metal Electrodes in Aqueous Electrolytes through an Operando Study,” *Energy Storage Materials* 46 (2022): 243-251. <https://dx.doi.org/10.1016/j.ensm.2022.01.012>

11	Z. Li, Y. Li, X. Ren, et al., “Elucidating the Reaction Mechanism of Mn^2+^ Electrolyte Additives in Aqueous Zinc Batteries,” *Small* 19 (2023): 2301770. <https://dx.doi.org/10.1002/smll.202301770>

12	V. Soundharrajan, B. Sambandam, S. Kim, et al., “The Dominant Role of Mn^2+^ Additive on the Electrochemical Reaction in ZnMn_2_O_4_ Cathode for Aqueous Zinc-Ion Batteries,” *Energy Storage Materials* 28 (2020): 407-417. <https://dx.doi.org/10.1016/j.ensm.2019.12.021>

13	M. Li, Q. He, Z. Li, et al., “A Novel Dendrite‐Free Mn^2+^/Zn^2+^ Hybrid Battery with 2.3 V Voltage Window and 11000‐Cycle Lifespan,” *Advanced Energy Materials* 9 (2019): 1901469. <https://dx.doi.org/10.1002/aenm.201901469>

14	K. A. G. de Alwis, Z. Chen, D. P. Guruge, et al., “Bilayered V_2_O_5_ Nanostrands with a PVA-Based Cathode Slurry for High-Performance and Sustainable Zinc-Ion Batteries,” *Green Chemistry* 28 (2026): 2519-2531. <https://dx.doi.org/10.1039/D5GC05207F>
